# Association between the use of free-of-charge intrauterine devices and a history of induced abortion: a retrospective study

**DOI:** 10.1186/s12905-019-0821-3

**Published:** 2019-10-18

**Authors:** Sabina Ulbricht, Angelika Beyer, Ulrich John

**Affiliations:** 1 Institute of Social Medicine and Prevention, University Medicine Greifswald, Greifswald, Walther-Rathenau-Str. 48, D-17475 Greifswald, Germany; 2 Institute for Community Medicine, Section Epidemiology of Health Care and Community Health, University Medicine Greifswald, Ellernholzstr. 1-2, D-17487 Greifswald, Germany; 3 Institute of Social Medicine and Prevention, University Medicine Greifswald, Greifswald, Walther-Rathenau-Str. 48, D-17475 Greifswald, Germany

**Keywords:** Contraception, Abortion, Unintended pregnancy, Family planning, Social welfare

## Abstract

**Background:**

To determine whether use of intrauterine device (IUD) is influenced by a history of induced abortion and the type of contraceptives used until costs are covered.

**Methods:**

We analyzed data from 301 female residents in Mecklenburg-West Pomerania, an economically challenged community. The women, aged between 20 and 35 years, were entitled to receive unemployment benefits, and had access to free-of-charge oral contraceptives, ring or IUD. Cross-sectional data were analyzed using logistic regression.

**Results:**

There were 112 (37.2%) women with a history of induced abortion, and 46 (15.3%) reported exclusively using less effective contraceptives (e.g. condoms). In a univariate logistic regression, use of an IUD was associated with a history of having had an induced abortion. Furthermore, uptake of an IUD was associated with women who had, until costs were covered, exclusively choice to use less effective contraceptives (OR = 3.281, 95% CI: 1.717; 6.273). Both associations remained significant in a multivariate model.

**Conclusions:**

Free contraceptives provided to women receiving unemployment benefits may increase the use of IUDs, especially among those with a history of an induced abortion and those using less effective contraceptives.

## Background

Unintended pregnancies are defined as those that are unwanted (because childbearing has been completed or no child is desired) or mistimed (those that have come earlier than desired) [1]. Unintended pregnancies account for 40% of pregnancies worldwide, resulting in 34 million unintended births and 42 million induced abortions per year [2]. Access to safe abortion represents a key component of public health initiatives that prevent death and disability among women due to pregnancy-related complications [3]. Previous studies from the United States of America (US) and Europe have shown that the likelihood of having an abortion is positively associated with such factors as having a lower educational level and lower income [4–6]. Data indicate that the number of unintended pregnancies can be reduced by offering all women access to effective methods of contraception [7]. Appropriate counseling by clinicians and other professionals should respect a woman’s autonomy in regards to her choice for a contraceptive method that is suitable for the specific reproductive stage of her life [8, 9].

The most effective methods are long-acting contraceptives, including intrauterine devices (IUDs) [7]. The use of IUDs after an abortion has the potential to decrease the number of subsequent unintended pregnancies and the attendant risks of induced abortions [9–11].

IUD use among women using contraceptives varies across European countries. In Eastern Europe, IUDs are used by over 25% of women in Moldova and Belarus. In Western European countries, such as Germany, Switzerland and the Netherlands, the percentage is less than 10% with the exceptions of France (18.9%) and Austria (15.4%) [12]. However, IUD use is influenced by the attitudes and experiences of clinicians, as well as by the knowledge, socio - cultural and religious environments of women [13]. Furthermore, IUD use is influenced by the financial costs of the device and its insertion, which may contribute to their low use when compared to similar or less effective but less expensive contraceptive methods, such as the oral contraceptive pill or condoms [14, 15]. Findings from a previous study conducted in the US indicated that IUD use increased after the introduction of a low-cost IUD (levonorgestrel 52 mg, Liletta®) [16]. Additionally, cost coverage was found to be associated with an increased use of IUDs too [17].

A study on fertility control and access to contraceptive methods in Europe found that none of the 16 countries investigated ensured full reimbursement for modern contraceptive methods and related health services [18]. In Germany, reimbursement for contraceptives is not available for women over 20 years old [18]. Among low-income women in Germany, especially those who receive financial unemployment benefits, were more likely either to abstain from contraceptive use in general or to fall back on less effective contraceptive methods, such as condoms [19].

We hypothesized that when IUD costs are fully covered, women with a history of induced abortion will be more likely to choose IUD placement compared to those without such a history. We further hypothesized that when IUD costs are fully covered, women who have been using less effective contraceptive methods, such as condoms or the calendar method, would be more likely to decide on IUD placement compared to those who have been using equally or less effective contraceptives such as the pill. We examined both hypotheses in a group of socially disadvantaged women who were entitled to receive unemployment benefits (Sozialgesetzbuch no. II), and had access to a selection of reversible, free-of-charge contraceptives, over the course of 12 month.

## Methods

### Participants and procedures

Study participants were women between the ages of 20 and 35 years, and were residents in one of the pre-defined urban or rural zip-code areas in the German Federal State of Mecklenburg-West Pomerania. All of them received financial unemployment benefits from a job agency. In Germany, job agencies are responsible for the implementation of basic security benefits for job seekers.

As part of the drive to focus attention on and uptake of the free-of-charge contraceptives, regional campaigns within the predefined zip-code areas were launched. Flyers and posters with a description of the program, inclusion criteria, and contact information were shared with professionals such as gynaecologists, pharmacists, counselors in pregnancy counseling services as well as with counselors in the job centers. The program was promoted multiple times via radio, newspapers, and television to reach the widest possible audience.

Women could choose between the oral contraceptive pill, the ring or the IUD. The visit of a gynaecologist was necessary to receive a prescription for anyone of the contraceptives. Further, the receipt of unemployment benefits among women interested in receiving free of charge contraceptives was verified at pregnancy counseling centres (rural area *n* = 2, urban area *n* = 2). The prescription for the selected contraceptive was marked with a notation for the pharmacy to deliver this free of charge. An offer was extended to discuss the contraceptive method chosen by the woman and her gynaecologist with a counselor in the pregnancy-counseling centre. All women completed an anonymous, self-administered, computerized survey during their appointment. Oral consent to participate was obtained from counselors in the pregnancy counseling centres. Data were collected between November 2013 and October 2014. All ethical aspects of the study were approved by the advisory board (3 gynecologists, 3 counselors from pregnancy counseling services, 1 pharmacist and 1 commissioner for data security) of the project and conducted in accordance with CONSORT guidelines. Administrative permission to conduct the study was given by the Ministry of Social Affairs, Integration, and Gender Equality of Mecklenburg-West Pomerania.

### Measures

The self-administered, computerized survey included questions about age, number of children, relationship status (living with a partner or not), educational level, region of residence (urban/rural), and the number of months unemployment benefits had been received (< 12, 12–36, > 36). The highest educational level attained was recorded under three categories: “< 10 years” (no graduation), “10 years” (secondary school certificate), or “> 10 years” (intermediate general school certificate or qualification for university entrance). The number of past abortions was classified into three categories: 0, 1, and > 1 (Additional file 1). We further assessed the type of contraceptive methods presently being used before the offer of free contraception. Effective contraceptive methods consisted of the pill, an IUD, subdermal implants or injectable hormonal contraceptives. Less effective contraceptive methods included the use of condoms, diaphragms, coitus interruptus, chemical methods (creams and suppositories), and fertility-awareness-based methods (the calendar, cervical mucus, and temperature methods) (Additional file 1). Only induced abortions that is, those that were intentional and carried out by a physician, were considered in this study.

### Data analysis

To characterize the study sample, data were reported as mean with standard deviations (SD) for continuous variables and as numbers and percentages for categorical variables. We examined the following factors known to be associated with IUD usage: age, socio-economic status (education, duration of receipt of unemployment benefits, and region of residence) [5, 6, 20, 21], and the exclusive use of less effective contraceptives until free-of-charge contraception was offered [21].

We conducted a series of univariate logistic regression analyses for these factors, as well as the number of past abortions. The dependent variable, a change of contraception method to an IUD, consisted of two categories: “Change to IUD” and “No change to IUD” (Reference category). A multivariate logistic regression analysis was performed to determine the effect of all variables when entered simultaneously. We used the multivariable fractional polynomial algorithm to test for non-linear effects of the continuous variable “age” [22]. To account for health care provider related concerns against offering the IUD to nulliparous women, we analyzed the data only among women who reported having had at least one live birth. From the total sample (*N* = 378) we removed nulliparous women (*n* = 70), as well as those who provided no information regarding their history of abortion (*n* = 7). The final sample comprised 301 women. Data were analyzed with Stata/SE version 14.2. A significance level of *p* < .05 was used in all analyses.

## Results

### Characteristics of the sample

Among a total of 301 women, the mean age was 27.4 years (SD = 3.84), 61.5% lived with a partner, and 61.8% had received unemployment benefits for at least 36 months. A number of 112 (37.2%) women reported a history of having had at least one abortion. Of these women, 68 (22.6%) reported a history of two or more abortions. The exclusive use of less effective contraceptives before free contraception was offered was reported by 15.3% of the women (Table 1).

**Table 1:** Sample characteristics

	Total (*n* = 301)
Age, Mean (standard deviation)	27.4 (3.84)
Relationship status: lived with partner	185 (61.5)
Educational (years of schooling)	
< 10	141 (47.2)
10	146 (48.8)
> 10 h	12 (4.0)
*No information (n = 2)*	
Receipt of unemployment benefits (months)	
< 12	29 (9.6)
12–36	86 (28.6)
> 36	186 (61.8)
Region of residence	
Rural	144 (47.8)
Urban	157 (52.2)
Number of induced abortions in the past	
0	189 (62.8)
1	44 (14.6)
> 1	68 (22.6)
Exclusive use of less effective contraceptive methods^a^	46 (15.3)

Note: Values are numbers (percentage) unless stated otherwise. ^a^Comprises the exclusive use of condom, diaphragm, coitus interruptus, chemical methods (cream, suppositories), and natural methods (calendar, cervical mucus, and temperature) until the coverage of costs and within the current period of receipt unemployment benefit

### Association between a change to an IUD, a history of abortion, and socio-demographics

Seventy-seven women (25.6%) changed to an IUD due to the free-of-charge option. Based on the univariate logistic regression analysis, this change was associated with a history of an induced abortion (1 abortion: OR = 3.430 [95% CI: 1.743; 6.753], > 1 abortion: OR = 1.663 [95% CI: 0.935; 2.954], Reference category: no past abortion). Women who exclusively used less effective contraceptives until costs were covered and who were currently receiving unemployment benefits were more likely to choose IUD placement (OR = 3.281 [95% CI: 1.717; 6.273], Reference category: use of effective contraceptives, e.g. oral contraceptive pill). No associations were found between changing to an IUD and socio-demographics such as age, relationship status, educational level, region of residence, or length of unemployment benefit receipt (Table 2). The association between changing to an IUD and a history of an induced abortion, along with the exclusive use of less effective contraceptives until costs were covered remained significant in the multivariate model, which included all variables simultaneously. No associations between changing to an IUD and age, relationship status, educational level, region of residence or length of unemployment benefit receipt was found in the multivariable model (Table 2).

**Table 2:** Univariate and multivariate-tested associations between change to IUD free of charge and the history of abortion and socio-demographics

	Logistic Regression					
	Univariate			Multivariate		
	OR	95% CI	*P-value*	OR	95% CI	*P-value*
Age group (years)	1.028	0.660; 1.128	0.925	1.042	0.967; 1.093	0.375
Relationship status: lived with partner	1.156	0.712; 1.878	0.556	1.388	0.808; 2.381	0.235
Education (years of schooling)			0.574			0.699
< 10	*Ref.*			*Ref.*		
10	1.102	0.680; 1.786		1.074	0.634; 1.819	
> 10	1.878	0.545; 6.131		1.769	0.472; 6.622	
Region of residence						0.062
Urban	*Ref.*			*Ref.*		
Rural	1.361	0.850; 2.180	0.198	1.640	0.975; 2.760	
Receipt of unemployment benefits (months)			0.134			0.124
< 12	*Ref.*			*Ref.*		
12–36	1.844	0.754; 4.510		2.042	0.766; 5.44	
> 36	1.111	0.478; 2.583		1.163	0.446; 3.032	
Number of induced abortion(s) in the past			0.001			0.003
0	*Ref.*			*Ref.*		
1	3.430	1.743; 6.753		3.307	1.602; 6.827	
> 1	1.663	0.935; 2.954		1.694	0.918; 3.121	
Exclusive use of less effective contraceptives^a^	3.281	1.717; 6.273	< 0.001	3.062	1.532; 6.119	0.002

^a^ Comprises the exclusive use of condom, diaphragm, coitus interruptus, chemical methods (cream, suppositories), and natural methods (e.g. calendar method, cervical mucus, and temperature) prior to the access of contraception free of charge and within the current period in receipt of unemployment benefit

## Discussion

Our study produced two main findings: First, changing to an IUD was associated with a history of an induced abortion. Second, there was an association between changing to an IUD and having used less effective contraceptives until the provision of free contraceptives.

Our study adds to growing evidence that when contraceptives are offered for free, women who have recently had an abortion [9–11], as well as those with a history of an induced abortion, will be more likely to choose IUD placement compared to women without such a history. Given the well-established finding that use of effective contraceptives, such as an IUD, have the potential to prevent unintended pregnancies, our results suggest that providing free contraceptives to women with low incomes, such as the unemployed, is an effective method to prevent unintended pregnancies. The proportion of 37.2% who reported a history of at least one abortion underscores the need to ensure access to contraceptives for women, irrespective of their costs.

As demonstrated in our study, it appears that socially disadvantaged women who use less effective contraceptives, such as condoms or calendar method, exclusively are more likely to benefit from the free provision of IUD placement compared to those who use more effective contraceptives, such as oral contraceptive pills.

Considering that socio-economically disadvantaged subpopulations are particularly difficult to reach for preventive measures, the proportion of 47.2% of women with no educational graduation in our sample is remarkable.

This study has a number of limitations. First, our results may not be generalizable to the female population as a whole. The use of free contraceptives was restricted to women aged between 20 and 35 years who received unemployment benefits. Nevertheless, the free-of-charge programme was directed to an important target group of German women, given that 72% of induced abortions in 2017 were carried out by those between 18 to 34 years of age [23]. Furthermore, access to the offer was restricted to those with residences in pre-defined urban or rural zip-code areas in the German Federal State of Mecklenburg-West Pomerania. Second, entry of women in the programme was restricted to 12 months. Thus, we were not able to evaluate the degree to which the IUD was used over time. Additionally, the free contraceptives being offered were restricted to the pill, IUDs and hormonal rings, which may have led to a selection bias among the sample. Furthermore, there was no information about the preferences of gynaecologists in regards to promoting the use of IUDs. The woman - gynaecologist interaction may be particularly important in how a contraceptive method is chosen [9], but discussing the reproductive intentions of women requesting contraceptive counseling appears to be challenging [8]. Third, the cross-sectional nature of the study design does not allow conclusions to be made regarding decreases in unintended pregnancies and a reduced number of abortion(s), as shown in previous studies [10, 11]. Fourth, there may be a bias in regards to the uptake of IUDs caused by the different socio - cultural and religious environments of the women. A proportion of 88.7% (*n* = 267) in our sample did not follow any religion, 8.6% (*n* = 26) were Protestants, and 2.7% (*n* = 8) were followers of other religions.

Despite these limitations, our study highlights the importance of free contraceptives, especially for methods with high initiation costs, such as IUDs.

## Conclusions

Contraceptives provided for free to women receiving unemployment benefits may increase the use of IUDs, especially among those with a history of abortion and among those who unavoidably use less effective contraceptives. There is an urgent need to advocate for comprehensive coverage of costs for contraceptives for all, to prevent unintended pregnancies as well decrease the abortion rate.

## Supplementary information

**Additional file 1.** Questionnaire about contraceptive methods used before the free of charge option. Questionnaire about induced abortions in the past.

## Data Availability

The data that support the findings of this study are available from the corresponding author on request. Researchers requesting the data will be required to sign a contract ensuring data usage in compliance with the statement given in the informed consent procedure and with the German data protection law, that the data will not be transferred to others, and that the data will be deleted after the intended analyzes have been completed.

## Abbreviations

CI: Confidence interval
IUD: Intrauterine device
OR: Odds ratio
Ref: Reference
SD: Standard deviation

## References

[1] Bexhell H, Guthrie K, Cleland K, Trussell J (2016). Unplanned pregnancy and contraceptive use in Hull and East Yorkshire. Contraception..

[2] Sedgh G, Singh S, Hussain R (2014). Intended and unintended pregnancies worldwide in 2012 and recent trends. Stud Fam Plan.

[3] Restricted access to abortion violates human rights, precludes reproductive justice, and demands a public health intervention; 2015. http://www.apha.org/policies-and-advocacy/public-health-policy-statements/policy-database/2016/01/04/11/24/restricted-access-to-abortion-violates-human-rights.

[4] Jones RK, Jerman J (2017). Population group abortion rates and lifetime incidence of abortion: United States, 2008-2014. Am J Public Health.

[5] Rasch V, Gammeltoft T, Knudsen LB, Tobiassen C, Ginzel A, Kempf L (2008). Induced abortion in Denmark: effect of socio-economic situation and country of birth. Eur J Pub Health.

[6] Vaisanen H (2015). The association between education and induced abortion for three cohorts of adults in Finland. Popul Stud (Camb).

[7] Gavin L, Moskosky S, Carter M, Curtis K, Glass E, Godfrey E (2014). Providing quality family planning services: Recommendations of CDC and the U.S. Office of Population Affairs. MMWR Recomm Rep.

[8] Skogsdal YRE, Karlsson JA, Cao Y, Fadl HE, Tyden TA (2018). Contraceptive use and reproductive intentions among women requesting contraceptive counseling. Acta Obstet Gynecol Scand.

[9] Roe AH, Bartz D (2019). Society of Family Planning clinical recommendations: contraception after surgical abortion. Contraception..

[10] Bednarek PH, Creinin MD, Reeves MF, Cwiak C, Espey E, Jensen JT (2011). Immediate versus delayed IUD insertion after uterine aspiration. N Engl J Med.

[11] Goodman S, Hendlish SK, Reeves MF, Foster-Rosales A (2008). Impact of immediate postabortal insertion of intrauterine contraception on repeat abortion. Contraception..

[12] World contraceptive patterns; 2013. http://www.un.org/en/development/desa/population/publications/pdf/family/worldContraceptivePatternsWallChart2013.pdf.

[13] Group ECW (2008). Intrauterine devices and intrauterine systems. Hum Reprod Update.

[14] Secura GM, Allsworth JE, Madden T, Mullersman JL, Peipert JF (2010). The Contraceptive CHOICE Project: reducing barriers to long-acting reversible contraception. Am J Obstet Gynecol.

[15] Dusetzina SB, Dalton VK, Chernew ME, Pace LE, Bowden G, Fendrick AM (2013). Cost of contraceptive methods to privately insured women in the United States. Womens Health Issues.

[16] Roth LP, Sanders JN, Simmons RG, Bullock H, Jacobson E, Turok DK (2018). Changes in uptake and cost of long-acting reversible contraceptive devices following the introduction of a new low-cost levonorgestrel IUD in Utah's title X clinics: a retrospective review. Contraception..

[17] Heisel E, Kolenic GE, Moniz MM, Kobernik EK, Minadeo L, Kamdar NS (2018). Intrauterine device insertion before and after mandated health care coverage: the importance of baseline costs. Obstet Gynecol.

[18] Barometer of Women’s Access to Modern Contraceptive Choice in 16 EU Countries; 2015. https://www.ippfen.org/sites/ippfen/files/2017-04/IPPF%20EN%20Barometer%202015%20contraceptive%20access.

[19] Helfferich C. Bezug staatlicher Sozialleistungen und Verhütung. FORUM Sexualaufklärung und Familienplanung. 2016:3–8.

[20] Jones RK, Jerman J (2017). Characteristics and Circumstances of U.S. Women Who Obtain Very Early and Second-Trimester Abortions. PloS one.

[21] Ulbricht S, Beyer A, John U (2018). The use of free-of-charge prescription contraceptives among women : results of a pilot project in the German federal state of Mecklenburg-Western Pomerania. Bundesgesundheitsblatt Gesundheitsforschung Gesundheitsschutz.

[22] Royston Patrick, Altman Douglas G. (1994). Regression Using Fractional Polynomials of Continuous Covariates: Parsimonious Parametric Modelling. Applied Statistics.

[23] DESTATIS: Statistisches Bundesamt. Schwangerschaftsabbrüche; 2018. https://www.destatis.de/DE/ZahlenFakten/GesellschaftStaat/Gesundheit/Schwangerschaftsabbrueche/Tabellen/Alter.html;jsessionid=3B9C063F7E9D83CBC666563A20CFB64A.InternetLive1%20.and.

