# Supplementary material for: Association between the use of free-of-charge intrauterine devices and a history of induced abortion: a retrospective study
Source: BMC Womens Health. 2019 Oct 18;19:120. doi: 10.1186/s12905-019-0821-3 (PMC6798342; doi:10.1186/s12905-019-0821-3)
Supplement: Supplementary file 1 — Additional file 1. Questionnaire about contraceptive methods used before the free of charge option. Questionnaire about induced abortions in the past. [file 12905_2019_821_MOESM1_ESM.docx]

**Additional file 1**

**Questionnaire**

**Which contraceptives have you used before the offer of free contraception?**

□ Pill

□ Subdermal implants

□ Ring

□ Condome

□ Intrauterine device (copper)

□ Intrauterine device (hormonal)

□ Stick

□ Injectable hormonal contraceptives

□ Diaphragms

□ Coitus interruptus

□ Chemical methods (creams or suppositories)

□ Fertility-awareness-based methods (e. g. the calendar, cervical mucus, and temperature methods)

□ Others:_____________________________________

**Have you ever decided to have an induced abortion?**

□ Yes □ No 🢂 *if no, please continue with question no …*

□ I do not want to give any details.

**How often have you decided to have an abortion in your life?**

| □ once |
| --- |
| □ two times |
| □ three times |
| □ four times |
| □ five times  □ more than five times |
